# Synteny mapping between common bean and soybean reveals extensive blocks of shared loci

**DOI:** 10.1186/1471-2164-11-184

**Published:** 2010-03-18

**Authors:** Phillip E McClean, Sujan Mamidi, Melody McConnell, Shireen Chikara, Rian Lee

**Affiliations:** 1 Genomics and Bioinformatics Program, North Dakota State University, Fargo, ND 58105, USA; 2 Department of Plant Sciences, Loftsgard Hall, North Dakota State University, Fargo, ND 58105, USA

## Abstract

**Background:**

Understanding syntentic relationship between two species is critical to assessing the potential for comparative genomic analysis. Common bean (*Phaseolus vulgaris *L.) and soybean (*Glycine max *L.), the two most important members of the Phaseoleae legumes, appear to have a diploid and polyploidy recent past, respectively. Determining the syntentic relationship between these two species will allow researchers to leverage not only genomic resources but also genetic data for important agronomic traits to improve both of these species.

**Results:**

Genetically-positioned transcript loci of common bean were mapped relative to the recent soybean 1.01 pseudochromosome assembly. In nearly every case, each common bean locus mapped to two loci in soybean, a result consistent with the duplicate polyploidy history of soybean. Blocks of synteny averaging 32 cM in common bean and 4.9 Mb in soybean were observed for all 11 common bean linkage groups, and these blocks mapped to all 20 soybean pseudochromosomes. The median physical-to-genetic distance ratio in common bean (based on soybean physical distances) was ~120 kb/cM. ~15,000 common bean sequences (primarily EST contigs and EST singletons) were electronically positioned onto the common bean map using the shared syntentic blocks as references points.

**Conclusion:**

The collected evidence from this mapping strongly supports the duplicate history of soybean. It further provides evidence that the soybean genome was fractionated and reassembled at some point following the duplication event. These well mapped syntentic relationships between common bean and soybean will enable researchers to target specific genomic regions to discover genes or loci that affect phenotypic expression in both species.

## Background

Comparative genetics and genomics leverages knowledge from companion species and attempts to define important evolutionary relationships. Important to understanding these relationships is the physical and genetic synteny between any two species. Physical synteny can be used to explain the cytogenetic events that a genome has undergone as it evolved along a lineage into its current structural form. Associated with these genomic events is the evolutionary repositioning of genes responsible for phenotypes shared between the two species. This repositioning places genes in different genomic contexts that could alter the degree and timing of their phenotypic effects. Understanding the overall positioning of genes in two related species can suggest strategies that aid the cloning of a gene in one species based on its position in a reference species.

Synteny has been studied quite extensively in plants, beginning with the early macro comparisons using RFLP markers. Although these analyses used a limited number of markers, they revealed major syntenic themes that have informed much of the research in the field. Closely related species, such as tomato and potato [1,2], have highly conserved marker order that is only disturbed by clearly defined events such as paracentric inversions. At greater evolutionary distances, as evidenced by tomato and pepper [3], inter- and intra-chromosomal translocations have redistributed loci such that only short syntentic blocks are observed while the chromosome number remains unchanged. Finally, a one-to-two mapping of loci, as seen with sorghum and maize [4], revealed the effects of polyploidy on synteny. These types of studies, involving multiple species and using collections of shared probes, have revealed major evolutionary relationships within lineages such as the grasses [5] and legumes [6]. For the grasses, using 30 segments of the rice genome as a reference, the genomes of ten other species could be reconstructed [5].

More recently, *Brassicaceae *evolution was studied in depth with a high density set of markers. Using the genome of the amphidiploid *B. napus *as a reference, the duplication history of its two parents, *B. rapa *and *B. oleracea*, revealed earlier rounds of duplication shared between these two diploid genomes and suggested that a progenitor with fewer chromosomes was responsible for the lineage [7]. Finally, a comparison of a dense genetic map of *B. napus*, consisting of > 1000 loci orthologous to Arabidopsis genes, and the genomic sequence of Arabidopsis, revealed that 90% of the *B. napus *genome could be reconstructed from 21 orthologous blocks of Arabidopsis genome while a number of the Arabidopsis segments were duplicated in the *B. napus *genome [8].

With the wide-spread availability of whole genome, BAC, and EST sequencing, and new approaches to high density mapping, further aspects of comparative evolution were revealed. Early comparisons of the genomic sequence data from Arabidopsis and tomato, two species whose divergent evolution began 94 MYA at the time of the Asterid/Rosid divergence [9], revealed a pattern of segmental duplication followed by local gene loss [10]. A similar pattern, but not to the same degree, was observed between Arabidopsis and *B. oleracea*, two species that diverged ~20 MYA [11]. The length of the shared block appears to depend upon the divergence time. The rice/wheat synteny appears to vary, with some regions showing extensive microcolinearity [12,13] while other regions exhibit a history of cytological events that has reduced the extent of local syntenty [14,15]. Maize and sorghum are recently related evolutionarily through a common ancestor that gave rise to sorghum and the two progeneitors of maize [16]. The species diverged only 12 MYA [17], yet the synteny between sorghum and maize appears to be less extensive than that between sorghum and rice that diverged ~40 MYA [18,19].

**Figure 1:**
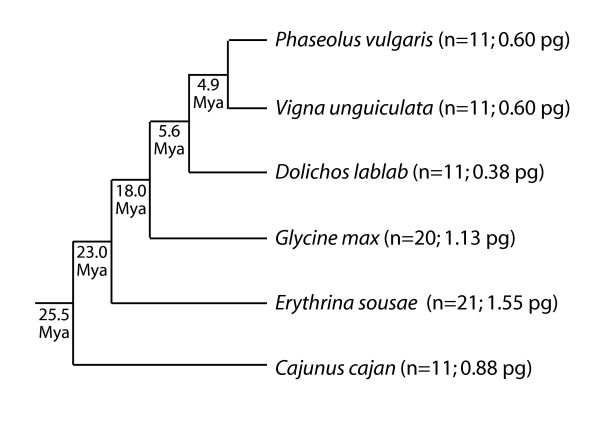
**Phylogeny of economically important Phaseoleae legumes**. The phylogeny is condensed from that presented in Stefanovic et al. (2009). The nodal dates are the minimum date of the range based on 32.1 million years ago (MYA) date of "A" node representing the divergence of the Phaseoleae legumes from *Apios americana*. The haploid chromosome number and C-value [in piocgrams (pg)] are from the Plant C-Value Database http://data.kew.org/cvalues/; verified May 5, 2009).

Here we investigate the syntenic relationship between two important legumes, common bean (*Phaseolus vulgaris *L.) and soybean (*Glycine max*). These two species are the two most important economically important legumes, soybean for its many human and animal usages, and common bean as an important nutritional crop for many economically poorer countries [20]. These two legume species are members of the Phaseoleae (Figure 1), a clade within the economically important Papilionoideae legumes. This clade diverged from the IRLC clade, the other economically important Papilionoideae clade, 54.3 MYA [21,22].

Common bean and soybean diverged 19 MYA [21,22]. Determining those genomic events that followed the divergence is important as investigators attempt to leverage the recently released soybean genome sequence [23] as a tool for research within the clade. RFLP mapping [24] and EST Ks analysis [25] provide strong evidence that soybean under went a major duplication event dated at 11 MYA [[25], Mamidi S, Lee RK, Terpstrea J, Lavin M, Schlueter JA, Shoemaker RC, McClean PE: Whole genome duplications in the evolutionary history of legumes, submitted]. Most likely this was an autopolyploid event [22,26]. By contrast, molecular marker data [27] show that common bean is a diploid while EST Ks analysis suggests that its genome has only undergone localized segmental duplications. Given their close relationship within the Phaseoleae, common bean is considered a diploid model for soybean [20]. Low-density RFLP mapping [28] found that the two species share a high degree of sequence homology but synteny is only found only over shorts blocks of the genomes. This is in contrast to the long stretches of synteny shared between common bean and mung bean (*Vigna radiate*), another member of the Phaseoleae clade. These two species diverged between 4.9 and 8.0 MYA [21,22].

To better understand the structural relationships between the common bean and soybean genome, we compared the organization of an extensive gene-based map of common bean with the complete sequence of the soybean genome. We discovered overwhelming evidence of a one-to-two relationship between common bean and soybean sequences. In addition, we were able to trace many of the gene rich regions of all soybean chromosomes back to specific regions of the common bean genetic map. Evidence is also provided that, relative to common bean, soybean is segmentally rearranged. Using this genetic/physical synteny, we have also been able to electronically map an additional 20,000 common bean EST contigs and singletons relative to soybean. This result is a major first step in understanding the evolution of the soybean genome relative to a diploid species within the Phaseoleae clade while providing a framework for the comparative genetics and genomics of these two species.

## Results and Discussion

A few limited studies have investigated the relationship between the common bean and soybean genomes. Boutin et al. [28] utilized shared RFLP markers and discovered a number of shared common bean/soybean syntentic markers blocks. In addition, individual markers showed a more complex pattern of syntenty between the two species suggesting a pattern of fragmentation in the evolutionary history of soybean relative to common bean. Subsequently, Lee et al. [29] built upon these results and were the first to show a clear one-to-two relationship between the common bean and soybean genomes. Although these results are compelling, they only provided a limited description of the genomic relationships between these two species. With a richer array of sequenced-based resources, including an increasing number of ESTs in common bean and an extensive draft sequence of the soybean genome, it is now possible to investigate the syntentic relationships between these two species at a greater depth.

### Common bean and soybean orthologous loci

Our first analysis of common bean and soybean synteny was based on 300 gene-based loci [McConnell M, Chikara S, Mamidi S, Rossi M, Lee R, McClean PE. A gene-based linkage map of common bean (*Phaseolus vulgaris*), submitted.] that were genetically mapped using the community-wide *P. vulgaris *BAT93 × Jalo EEP558 mapping population and 59 gene-containing RFLP probes [30,31]. These loci were compared to the first public release of the soybean genome, consisting of 20 pseudochromosomes, using the blastn algorithm. We first limited the hits to those with an E-value < 1 × 10^-10^. This decision was motivated by our decision to compare the two genomes by uncovering those soybean loci with some degree of orthology to the common bean query sequences. A total of 1065 hits were detected that met this criterion, and the median E-value for these hits was 2.0 × 10^-61^. These hits were to loci on all 20 soybean pseudochromosomes.

**Figure 2:**
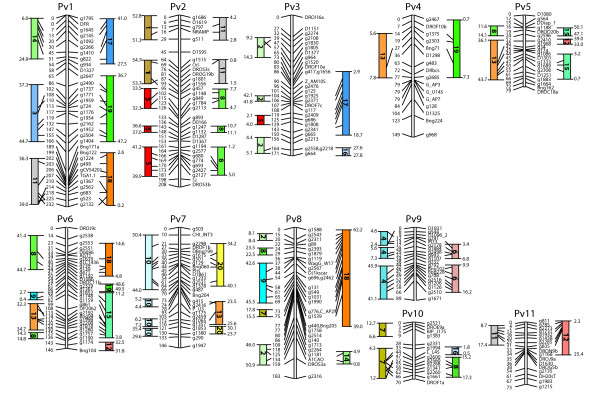
**Syntentic relationships of soybean relative to the common bean genetic map**. A common bean genetic map anchors corresponding syntenic regions of the soybean 1.01 genome build. The location (in megabase pairs) of each soybean fragment straddling the common bean linkage is noted at the beginning and end of the homology.

Since another goal of this analysis was to determine the genomic relationships between common bean and soybean genomes, we next limited the analysis to the best two soybean hits for each common bean sequence. This decision was based on the assumption that soybean underwent a whole genome duplication event since it divergence from common bean, an assumption supported by a number of lines of evidence [24,25]. Therefore, absent a major reduction in newly duplicated soybean genes, there should be a one-to-two locus correspondence between common bean and soybean. Under this criterion, a total of 720 hits were observed, and the median E-value was 3.5 × 10^-91^. Again, hits to loci on all 20 pseudochromosomes were observed. A common bean centric display of the synteny is found in Figure 2.

### Conserved common bean and soybean genomic blocks

A conserved syntenic block was defined as one that shared three loci between common bean and soybean and covered a minimum of 4 cM of the common bean genetic map. A total of 55 syntenic blocks were observed between the two species. On average, each block consisted of 7 loci. From a common bean genetic perspective, the mean size of each syntenic block was 32 cM (with a median of 29 cM). 75% of the blocks were > 20 cM in length. Conversely, the mean physical distance relative to soybean was 4.9 Mb (with a median of 2.6 Mb). 63% of the blocks were > 2 Mb in length.

By comparing the location of these blocks, it is very clear that nearly all segments of the common bean genome mapped to two segments of the soybean genome. 69.3% of the genetic distance of the common bean map mapped to some region of the soybean genome. Much of the uncovered regions in common bean fell in larges gaps on the linkage map. Conversely, these blocks consisted of 271 Mb or 27.9% of the soybean genome sequence. To better understand the difference in genome coverage, we needed to integrate additional soybean genomic information into our analysis.

**Figure 3:**
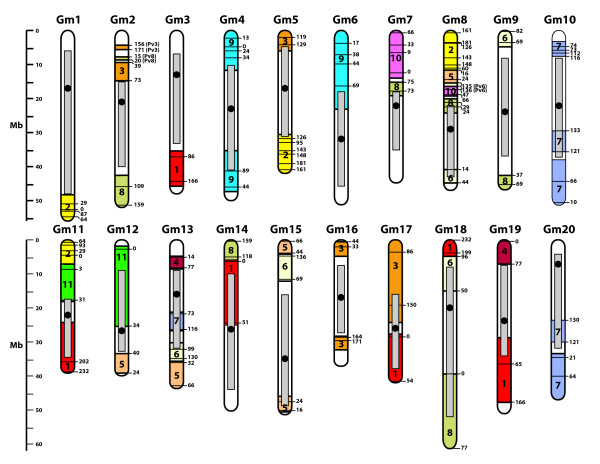
**Syntentic relationships of common bean relative to pseudochromosomes defined in build 1.01 of the soybean genome**. Painted on each soybean pseudochromosome are syntenic common bean fragments. The genetic location (in cM) of the fragment are noted. The extent to which each fragment was electronically extended (see text for procedure) beyond the genetic mapping distance is noted as the distance beyond the genetic boundaries of the syntenic common bean fragment. The gray bar represents the heterochromatic region, and the black dot is the location of the centromere. This information was collected from the Soybean Genome Browser at SoyBase.org http://soybase.org/gbrowse/cgi-bin/gbrowse/gmax1.01/.

As displayed graphically at Soybase http://soybase.org and Phyotzome http://www.phytozome.org/soybean, and captured in Figure 3, much of the soybean genome consists of pericentromeric DNA. Several features of this class of DNA are important for this analysis here. First, this DNA is centered around the centromeres and encompasses 56% of the soybean genome. Further, this DNA has a much lower gene density than euchromatic regions at the ends of the chromosomes. Finally, these regions have a much lower recombination rate than the euchromatic region. All of these features appear to be common for eudicot genomes.

As Figure 3 shows, 35 of the 40 of the soybean chromosome arms exhibit a signature of conserved synteny between the two species. Much of the synteny that we observed here was to orthologous loci in the euchromatic regions of the soybean genome. Only a few soybean chromosomes, specifically 10, 12, 14, 17, 18, and 20, contain extensive blocks of common bean loci that map to soybean pericentromeric DNA. If we exclude the pericentromeric DNA from our calculations, of the 271 Mb of the soybean that were syntentic to common bean, 200 Mb mapped to the euchromatic arms. Therefore, using this limited set of common bean loci, we were able to determine the ancestry of 42.7% of the gene rich euchromatic region of soybean. Further, 30 of the 33 duplicated blocks of soybean genome anchored to the common bean genetic map were also defined as soybean-to-soybean duplicates based on dot blot analysis of the full genome sequence of soybean [[23]; see Figure S5 there].

We next compared the ratio of the physical distance to genetic distance in common bean. To calculate the physical distance per cM, we used the physical distance in soybean relative to the genetic distance in common bean. This same approach was used when the *A. thaliana *and *B. napus *genomes were compared [8]. The physical distance per cM was calculated for a total of 245 comparisons of neighboring loci. The average distance was 290,441 bp/cM. The median ratio was 119,405 bp/cM, and 42% of the comparisons were less than 100,000 bp/cM. These later values were very similar to those observed when *A. thaliana *and *B. napus *were compared.

Next, a comparison of the physical to genetic distances between duplicate blocks from the soybean genome that were syntentic to the same common bean genetic block was made. The average difference between these block was 33,571 bp/cM, while the median was 18,056 bp/cM. The range of difference spread from 76 to 203,002 bp/cM. This largest difference was between duplicates on soybean chromosomes 8 and 18 that were syntenic to a Pv6 block bounded by markers g2553 and g139 in the interval from 23-47 cM. The ratio for these two blocks (Gm8 = 119,914 bp/cM; Gm198 = 351,548 bp/cM) was much greater than the genome-wide average. These two soybean blocks terminate in the low recombination region, and the Gm18 blocks goes further into the pericentromeric region. This probably accounts for the differences in the physical to genetic distance ratio.

### Electronic mapping of common bean sequences

Given the extensive synteny observed between common bean and soybean, we considered the possibility of mapping other common bean sequences relative to the observed duplicate syntentic blocks. The underlying concept is that duplicate soybean blocks could serve as a reference that would point to the most likely genetic position for any given common bean sequence. This concept is analogous to the binning of ESTs in wheat [32] except rather relying on the physical deletion landmarks it relies upon the comparative genomic structure between two species within the same evolutionary lineage. These electronically mapped loci might also 1) reveal additional details about the degree of synteny and the chromosomal history of these two species, and 2) point to duplication blocks in the soybean genome itself.

To test this concept, we collected all available common bean sequences for a blastn analysis. This analysis consisted of 11,043 EST contigs and 9,847 EST singletons that were constructed here using the procedure described by Childs et al. [33]; 85,102 repeat masked BES [34]; and 694 CDSs from common bean. Several criteria were used before a sequence was included in our set of electronically mapped sequences. First, the sequence must have two hits against different segments of the soybean genome. Secondly, the e-value for all hits must be less than 1 × 10^-30^. Next, the length of the match of the query to the soybean must be 150 nt or greater. And finally, to ensure that the homology was against two different sequences, the distance between the 3' end of one sequence and the 5' beginning of is neighboring sequence must be greater than 50 nucleotides. Using these criteria an additional 15,091 common bean sequences were electronically mapped. This consisted of 549 gene-based sequences, 2,548 BES, 4,522 EST singletons, and 7,472 EST contigs. Of these, 316 were previously mapped genetically. This gives a total of 14,775 newly mapped loci. The median distance between any two sequences, based on their soybean orthologous location, was 11,068 nucleotides.

In addition to its usefulness for studying common bean and soybean synteny, this large collection of electronically mapped common bean sequences greatly increases the marker set available for common bean genetics. For this to be the case, it was essential to determine if the electronic map position of each of these sequences corresponds to its predicted genetic position. To test this concept, we focused on a 43 cM region of Pv7 between markers g2298 and g1378. We selected this region because although QTL for common bacterial blight [35] and white mold resistance [36] are known to map to this location, useful markers have not yet been developed for marker assisted selection. Primers were designed to 15 EST contig or singleton sequence loci within the interval, and the amplification products from BAT93 and Jalo EEP558 were sequenced. Of these we detected 7 polymorphic loci. CAPs markers were developed, and the loci were mapped on the BAT93 × Jalo EEP558 community mapping population. All 7 of the loci mapped within the interval that was selected for study. Five of the seven mapped exactly as predicted. The positions of the remaining two loci were inverted relative to the positions of the two soybean orthologs. This result strongly suggests that the electronic mapping approach provides extensive physical and genetic position information not previously available for common bean.

These results allowed us to systematically extend the distance of the common bean syntenic block (Figure 3). The principle we applied is to search for a common bean genetic block shared by two soybean regions and extend the borders in either or both directions until sequence colinearity between the two soybean blocks is broken. For example, the Pv 9 block from 44-89 cM is shared with both Gm4, from positions 41.1 to 45.9 Mb, and Gm6, from positions 11.9 and 16.2 Mbp. By applying the principle described above, we see an unbroken block in the same orientation from 36.0-49.0 Mbp on Gm4 and from position 8.6-19.5 Mbp on Gm6. By applying this concept, we were able to extend the degree of synteny relative to this one Pv9 block from 9.1 Mbp to 23.1 Mbp. We reexamined the synteny between the two species and applied the same principle we used with the Pv9 to the entire genome. Using this newly derived information, we can account for an additional 187 Mbp of syntentic regions between common bean and soybean. All of our approaches here allow us to trace the ancestry of 456 Mbp of the soybean genome relative to the diploid common bean genome.

### Duplication history of soybean

Given that soybean has undergone a major duplication event in its history, it is reasonable to expect that many common bean sequences should map to two locations in soybean. Our extensive Blastn analysis using all available common bean sequences clearly showed this to be the case. Given that common bean is a diploid relative of soybean, the common bean sequences can then be used as a reference point that links two duplicate regions in soybean. This approach does have limitations, although not serious. First, any sequence or sequence block unique to the soybean lineage will not have a common bean sequence signal, and any duplication associated with those sequences will not be uncovered. Also, given that 83% of the sequences we were able to map electronically were gene-based, and that most genes are found in the euchromatic regions near the ends of eukaryotic chromosomes, the strongest evidence regarding the duplication history will come from those gene-rich regions.

To investigate the duplication history of each soybean chromosome relative to the remainder of the soybean chromosome set, we first ordered all of the common bean sequences relative to their soybean ortholog on a chromosome-by-chromosome basis. That ordering was cross-referenced to the duplicate with the lowest E-value on a second soybean chromosome. These defined blocks of loci that mapped to the same duplicate chromosome. It was required that each block consist of ten common bean loci, and that the loci must be in consecutive order.

**Figure 4:**
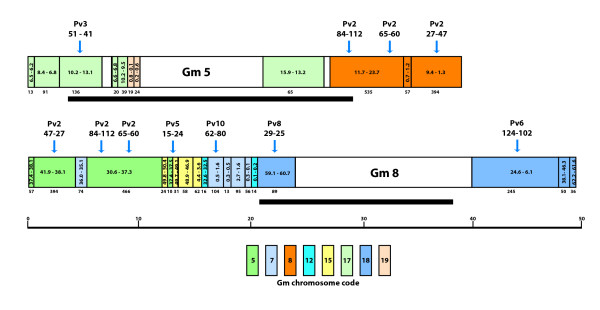
**Duplicated blocks along soybean chromosomes Gm5 and Gm8 based on reference ordering of common bean sequences**. Each chromosome consists of a series of duplicate blocks that are color coded. White blocks have no reference common bean sequence. The physical position (in Mbp) of the duplicate block on its home chromosome is given. The position is given in either direct or inverted order based on its position relative to the reference chromosome. Above some blocks is the genetic location of the corresponding syntentic common bean blocks. Below each block is the number of common bean sequences used to establish the block. The thin bars represent the location of the pericentromeric regions of the chromosomomes. The physical distance scale at the bottom is in Mbp.

Figure 4 summarizes the duplicated block data for chromosomes Gm5 and Gm8 in graphical form. These two were chosen because they are representative of the other chromosomes, and because they share synteny to the same regions on common bean Pv2. The most apparent observation is the modular nature of each chromosome with regards to their duplicate blocks. Gm5 shares duplication blocks with three other soybean chromosomes (Gm8, Gm17, Gm19) while Gm8 shares duplication blocks with Gm5, Gm7, Gm12, Gm15, and Gm18. Furthermore, the modular blocks from the duplicate chromosome are not contiguous. For example, beginning at position 0 on Gm8, there is about 12 Mbp of sequences duplicated on Gm5 in three non-contigous blocks. These blocks are interrupted by a duplicate block from Gm7. Two of the blocks on both Gm5 and Gm8 are syntenic to common bean Pv2. Furthermore, of the two chromosomes, Gm8 shares duplications with more Gm chromosomes which in turn trace back to five different common bean linkage groups.

Using both the Pv/Gm syntentic data as well the duplications found in Gm using the large data set from Pv, several conclusions can be drawn regarding the chromosomal history of soybean. First, the one-to-two mapping of Pv to Gm sequences provides further compelling evidence that a major duplication event is part of the history of the soybean genome [25]. The modular nature of the both the Pv/Gm synteny and soybean duplications suggest that either coincident with the duplication, or shortly after, the duplicated chromosomes were fractionated or new chromosomes were reassembled. A dramatic example is found by comparing the Gm15 duplicates to Gm8. Here we see three duplicate blocks from the end of Gm15 organized non-contiguously. These are right next to a block from the beginning of the same chromosome. A chromosome-wide analysis suggests that at least 19 blocks rearranged to form Gm8. Because of a lack of significant sequences homologous much of the pericentromeric region, we could not determine the modular nature of that region.

## Conclusions

We have used the first public release of the soybean genome to evaluate the syntentic relationship between this important economic species and common bean, another member of the Phaseoleae legumes. It appears that extensive regions of synteny exist between these two species. These relationships further suggest that the soybean genome has undergone a whole genome duplication based on the fact that nearly all of the common bean sequences that map to a single location, have two copies in the soybean genome. Furthermore, soybean appears to have undergone extensive chromosome breakage and rearrangement. This conclusion is based on the observation that most soybean chromosomes consist of fragments from multiple common bean chromosomes. Whether these rearrangements occurred prior to, coincident with, or following the duplication event is unclear at this time. From an applied perspective, these results suggest that a comparative genomics approach to gene discovery is feasible for these two evolutionarily related species.

## Methods

### EST contiging and BAC-end sequence processing

The 83,448 *Phaseolus vulgaris *sequences in the National Center for Biotechnology Information (NCBI) EST database available on August 1, 2008 were downloaded from http://www.ncbi.nlm.nih.gov/sites/entrez?db=nucest. EST contigs and singletons were defined using the procedures described in Childs et al. [33]. The procedure was performed using the following stand-alone software: SeqClean (http://www.tigr.org/tdb/tgi/software; default parameters), Megablast [[37]; http://www.ncbi.nlm.nih.gov/blast/megablast.shtml; default parameters], and CAP3 [[38]; default parameters]. All 89,017 BAC-end sequences (BES) were downloaded from the NCBI GSS database http://www.ncbi.nlm.nih.gov/sites/entrez?db=nucgss on August 1, 2008. We limited our analysis to those 85,102 BES representing sequences from both ends of 42,551 sBACs. Prior to the blastn analyis, these BES were analyzed using RepeatMasker (http://www.repeatmasker.org; default parameters, v. 3.15) with the Fabaceae repeats database obtained from Plant Repeat Database [[39]; http://plantrepeats.plantbiology.msu.edu/; file: TIGR_Fabaceae_Repeats.v2_0_0.fsa.txt]. A fasta file containing the singletons and EST contigs is available upon request from the corresponding author.

### Blastn analysis

The 20 pseudochromosomes from the version 1.01 release of the soybean assembly were downloaded from ftp://ftp.jgi-psf.org/pub/JGI_data/Glycine_max/Glyma1/assembly/sequences/ and used as a database for all blastn analyses. First, 300 EST or singletons, originally developed by Ramirez et al. [40], using a set of 21,026 EST, and 15 other sequences, all mapped onto the 11 common bean linkage groups at a LOD value of 2 [McConnell M, Chikara S, Mamidi S, Rossi M, Lee R, McClean PE. A gene-based linkage map of common bean (*Phaseolus vulgaris*), submitted.], were used as a query. Subsequently, blastn analyses were performed using the new set of ESTs (11,043) and singletons (9,847) as one query, and the 85,102 BES as a second query. A final query consisted of 694 complete *P. vulgaris *coding sequences (CDS) downloaded from NCBI http://www.ncbi.nlm.nih.gov/ on August 8, 2008. Because we were looking for significant levels of orthology between common bean and soybean sequences, we initially limited all blastn analysis to hits with E-values less than 1 × 10^-10 ^and overlaps of at least 150 nt. The results reported here are based on the first high scoring pair. See Additional File 1 for all of the common bean sequences that met these criteria.

### High density mapping of linkage group Pv7

Primers were developed for 15 contig or singletons that electronically mapped between markers g2298 and g1378 on linkage group Pv7. A 3'-primer was designed to a sequence within 150 nt of the putative stop, and the corresponding 5' primer was located about 500 nt upstream of the 3'-primer site. Subsequent PCR amplification and sequencing followed the procedures described in McClean et al. [41]. Based on sequence polymorphism, either CAPs or SNP diagnostic markers were developed. These markers were used to score polymorphisms among members of the community-wide BAT93 × Jalo EEP558 RI population. These marker loci were then mapped using the MAPMAKER software [42]. The final order was verified using the "ripple" command with a LOD value of 3.0.

## List of abbreviations

BAC: bacterial artificial chromosome; BES: BAC end sequence; bp: base pairs; CAPs: cleaved amplified polymorphic sequence; CDS: coding sequence; cM: centimorgans; EST: expressed sequence tags; Gm: *Glycine max*; LOD: logarithm of (base 10) of odds; Mb: megabases; MYA: million years ago; RFLP: restriction fragment length polymorphism.

## Authors' contributions

PEM conceived the project and was the principal author of the manuscript. MM performed all of the molecular marker analysis for common bean and analyzed the initial syntenty between the two species. RL and SM jointly developed the common bean contigs, and performed all of the bioinformatic analysis of blast analyses between the common bean sequences and the soybean 1.01 build. SC remapped the common bean molecular segregation data as new data became available. MM, RL, SM, and SC each reviewed the manuscript.

## Supplementary Material

Additional file 1 **Electronically mapped common bean loci based on synteny to the soybean genome sequence**. List of *P. vulgaris *loci which met the following selection criteria for electronic mapping: e-value less than 1 × 10^-30^, hits to two soybean chromosomes, query length greater than 149 nt, and soybean position for the end of one soybean locus and the beginning of the next locus was less than 50 nt. Click here for file
